# The Effect of Head Movements on Front-Back Discrimination and Sound Externalization With Hearing Aids

**DOI:** 10.1177/23312165261480035

**Published:** 2026-08-27

**Authors:** Tobias Greif, Virginia Best, Robert Baumgartner

**Affiliations:** 1 Acoustics Research Institute, Austrian Academy of Sciences, Vienna, Austria; 2 Institute of Computer Science; Faculty of Science, P. J. Šafárik University, Košice, Slovakia; 3 Department of Speech, Language and Hearing Sciences, 1846 Boston University, Boston, USA

**Keywords:** hearing aids, spatial hearing, externalization, self-motion

## Abstract

Head movements are intuitive, often spontaneous, behaviours that help us localize the origin of a sound source. In normal-hearing listeners, head movements during sound presentation are known to improve localization performance in both horizontal and vertical dimensions, as well as front-back discrimination. The perceived externalization of sound sources also improves with head motion. Hearing-aid users, especially users with behind-the-ear microphone positioning, exhibit diminished front-back localization abilities as well as degraded externalization under static conditions. The extent to which active head movements can overcome the spatial degradations introduced by hearing aids remains unclear. The current study aimed to provide quantitative data on this issue. Participants with normal hearing were fitted with low-gain hearing aids and presented with speech stimuli from a loudspeaker directly in front or behind. They performed front-back discrimination, rated perceived externalization, and did so with or without head movements. We hypothesized that head movements would fully resolve front-back confusions and restore externalization ratings to unaided levels. Instead, head movements were more effective in resolving front-back confusions than externalization breakdowns. We conclude that hearing-aid related spatial distortions may not be fully overcome by active head movements and note large inter-individual differences in the relationship between externalization and front-back discrimination.

## 1 Introduction

Due to the increasing prevalence of hearing-related difficulties world-wide (World Health Organization, 2025), a growing body of the population is using hearing aids (HAs). For instance, from 2011 to 2022, HA usage among older adults ( > 65 years) in the U.S. increased by 45.5% (Bessen et al., 2024). While HA users report and experience improvements in a variety of outcomes such as speech perception, communication function and hearing handicap (see Tang et al. (2025) for a recent review), they often still have notable difficulties with the spatial processing of sound (Akeroyd & Whitmer, 2011).

Some of these difficulties are related to the underlying hearing loss. Compared to listeners with normal hearing (NH), listeners with hearing impairment (HI) are less accurate at localizing sound sources in the horizontal and vertical dimensions, and are more likely to make front-back confusions (Best et al., 2010). On top of this, some studies that compared unaided and aided localization performance in HI listeners reported a detrimental effect of HAs (Byrne & Noble, 1998; Noble & Byrne, 1990). The potentially disruptive effect of HAs on sound localization is further evidenced by the fact that NH listeners fitted with HAs also exhibit impaired performance, especially for vertical plane localization and front-back discrimination (Best & Roverud, 2024; Denk et al., 2019; Noble & Byrne, 1990). These effects are in part related to the position of the HA microphone: behind-the-ear (BTE) HAs are more disruptive than in-the-ear (ITE) or completely-in-canal (CIC) devices that better capture pinna-related spectral cues (Best et al., 2010; Denk et al., 2019; Van den Bogaert et al., 2011). Strikingly, front-back discrimination can be at, or close to, chance-level under static listening conditions in HA users fitted with BTE devices (Best & Roverud, 2024; Keidser et al., 2006; Mueller et al., 2012; Van den Bogaert et al., 2011).

Recent work has identified another aspect of spatial perception that may be disrupted by HAs. HA users sometimes report issues with the “externalization” of sound sources, that is, under certain conditions they fail to successfully associate the sound image with its external source and instead feel like it is originating from within the head or at least closer to the head than it truly is (Best & Roverud, 2024; Boyd et al., 2012; Cubick et al., 2018; Noble & Gatehouse, 2006). This has been attributed to a number of possible factors, including ear canal occlusion, non-linear processing and disrupted pinna-related spectral cues (Baumgartner & Majdak, 2021; Best & Roverud, 2024; Byrne et al., 1998; Byrne & Noble, 1998; Cubick et al., 2018; Hassager et al., 2016, 2017). Early conceptual models of sound externalization suggest that externalization depends on a set of sensory cues that need to be consistent with those a real external sound source would provide in the environment, invoking also the notions of realism, validity, or plausibility (Durlach et al., 1992; Hartmann & Wittenberg, 1996). Baumgartner and Majdak (2021) translated this concept into a computational template matching framework, which was able to explain a variety of stimulus effects under anechoic static listening conditions based on a fixed weighting of monaural and interaural spectral shape cues. In this framework, incoming auditory cues are compared with memorized listener-specific spectral-shape templates. If the sensory input matches the established template, a successful externalization percept arises. If the input does not match, externalization perception breaks down. Similarly, contemporary models of directional sound localization also assume an ongoing comparison between incoming spectral-shape cues and established listener-specific templates. In particular, the saggital-plane localization model proposed by Baumgartner et al. (2016) is functionally largely equivalent to the externalization model (Baumgartner & Majdak, 2021), but the externalization model also considers the contribution of interaural spectral cues in addition to monaural spectral cues, which would suggest a partial, but not perfect correspondence between the two percepts. When listeners are fitted with HAs, there are multiple ways in which the incoming spectral-shape cues (both monaural and interaural) are disrupted. Depending on the exact devices and their fit on a specific listener, this may lead to a template mismatch and, thus, a breakdown of externalization and increased front-back ambiguity, as has been reported in previous work (Best et al., 2010; Best & Roverud, 2024; Denk et al., 2019; Noble & Byrne, 1990).

Under natural listening conditions, the human auditory system is rather well equipped to resolve front-back ambiguities. For example, by moving one’s head, dynamic interaural time and level differences are introduced that can help resolve confusions, as has been demonstrated for both NH and HI listeners (Brungart et al., 2017; Gessa et al., 2022; Higgins et al., 2023; Iwaya et al., 2003; Kato et al., 2003). Similarly, active head movements also improve the externalization of sound sources (Brimijoin et al., 2013; Hendrickx et al., 2017), with benefits that appear to persist even after the movement is terminated (Colas et al., 2023). It has been shown that *source* movements can improve perceptual externalization as well, but to a lesser extent than *head* movements (Hendrickx et al., 2017), suggesting that, in addition to the introduction of dynamic cues, active control and sensory predictability also play a role (Best et al., 2020).

Of primary interest in the current study is the relationship between front-back ambiguity and sound externalization. The observation has been made previously (Best et al., 2020; Durlach et al., 1992) that breakdowns of externalization and front-back confusions tend to occur under similar conditions and that one may in fact be a consequence of the other. For example, if a listener experiences uncertainty about whether a source is in front or behind, or perhaps perceives a source as simultaneously in front and behind, they may be unable to assign a reliable external location and hence may determine or respond that it is within the head. Similarly, if they fully internalize the sound image, it follows that they may be unable to determine its location in real space. While we know of no data directly addressing this relationship, there exists some indirect evidence that suggests one. Recently, Best and Roverud (2024) noted a relationship between rates of front-back confusion and breakdowns of externalization across different HA conditions in both NH and HI listeners. Specifically, they demonstrated that the breakdown of externalization was strongest in conditions with BTE microphones and strongly occluding power domes, which was also the condition yielding the highest rate of front-back confusions. However, this observation was made very broadly and at a group level, and the relationship between these two phenomena and its magnitude is not yet fully resolved.

The current study aimed to focus on this issue by jointly assessing externalization perception and front-back discrimination under different conditions in NH listeners. These conditions were chosen to vary the extent to which front-back confusions were expected, based on the extensive literature on this topic, and we then looked for concomitant changes in externalization. These conditions included BTE and ITE HAs (with ITE HAs expected to cause fewer front-back confusions) and head still vs. head moving (with head movements expected to eliminate front-back confusions). We hypothesized that if externalization breakdown is related to front-back ambiguity, manipulations that resolve either ambiguity should also restore the other. Alternatively, if front-back ambiguity and poor externalization are independent consequences of wearing HAs, resolving front-back ambiguities may not necessarily improve externalization. And, lastly, if front-back ambiguity only contributes partially to the externalization breakdown, we would expect an improvement in externalization with head movements but not a full restoration.

## 2 Methods

### 2.1 Participants

We recruited 18 young adults (16 female; mean age: 21.05 ± 2.18 years) with normal hearing, as assessed by audiometric thresholds of 20 dB HL or better at octave frequencies from 250 to 8000 Hz in both ears. Upon arrival in the lab, they provided written informed consent, completed an audiogram measurement, were fitted with the two sets of HA devices and then completed the experimental procedure described below. After the experiment had concluded, participants were informed in more detail about the purpose of the study. Total testing time was around 90 minutes.

### 2.2 Room and Equipment

The experiment took place in a single-walled sound booth (Industrial Acoustics Company) with interior dimensions of 3.8 × 4.0 × 2.3 m (length × width × height), with perforated metal walls and ceiling and a carpeted floor. The reverberation characteristics are described in detail in the ”BARE” room condition of Kidd et al. (2005). The participant was seated on a chair and stimuli were presented via two loudspeakers (Acoustic Research 215PS) located at ear level (0° elevation) directly in front of (0° azimuth), and directly behind (180° azimuth) the participant at a distance of 1.5 m. The loudspeakers were driven by a Lenovo PC, a multichannel soundcard (MOTU 16A), and a bank of power amplifiers (Crown Audio XTi 1002) that were all located outside of the booth. The room was dimly lit such that the loudspeakers were clearly visible to participants. Responses were collected using a handheld backlit keypad.

The HAs used for the experiment were two separate pairs of GN ReSound One receiver-in-the-canal HAs with Microphone and Receiver In-The-Ear (M&RIE) technology (Device ID/firmware version: RS.D2.Top.CbRIE45DW/9.60.1.1). These devices have the ability to switch between using either microphones on the body of the aid (”BTE”), or using a microphone on the outward facing component of the receiver worn in the ear (”ITE”), which allowed us to configure one set of HAs per condition. Both sets of HAs were set to have 10 dB of linear (compression ratio = 1) gain across all frequencies. Only mild feedback cancellation was turned on and all noise reduction and other features were turned off. Coupling to the ear canals was achieved via highly occluding power domes. Further details on the devices and fitting procedure can be found in Best and Roverud (2024). Our conditions closely followed the conditions ”BTE Closed” and ”ITE Closed” described there.

For headtracking, one lightweight inertial sensor (Movella DOT; ± 2000°/s gyroscope; ±16g accelerometer; ±8 Gauss magnetometer) was paired to a smartphone (Motorola moto g 5G) running the recording software (Movella DOT application, Version 6.1). The sensor was mounted on top of the participant’s head via a head strap and subsequently aligned to the frontal loudspeaker. Head motion data (Euler angles and free acceleration) was collected at a 60 Hz sampling rate in live streaming mode continuously during each individual block.

### 2.3 Stimuli and Procedure

Stimuli were monosyllabic words (e.g. “cold”; “new”; “bags”) spoken by 11 male and 11 female talkers, taken from a matrix-style corpus recorded at Boston University (Kidd et al., 2008). On each trial, a random word (from 32 unique words) and a random talker was chosen and presented from one of two locations (frontal vs. rear loudspeaker) at one of three presentation levels (50, 55 and 60 dB SPL). The minimum duration of a stimulus was 303 ms and the maximum 1110 ms, depending on the chosen talker and word.

**Figure 1.**
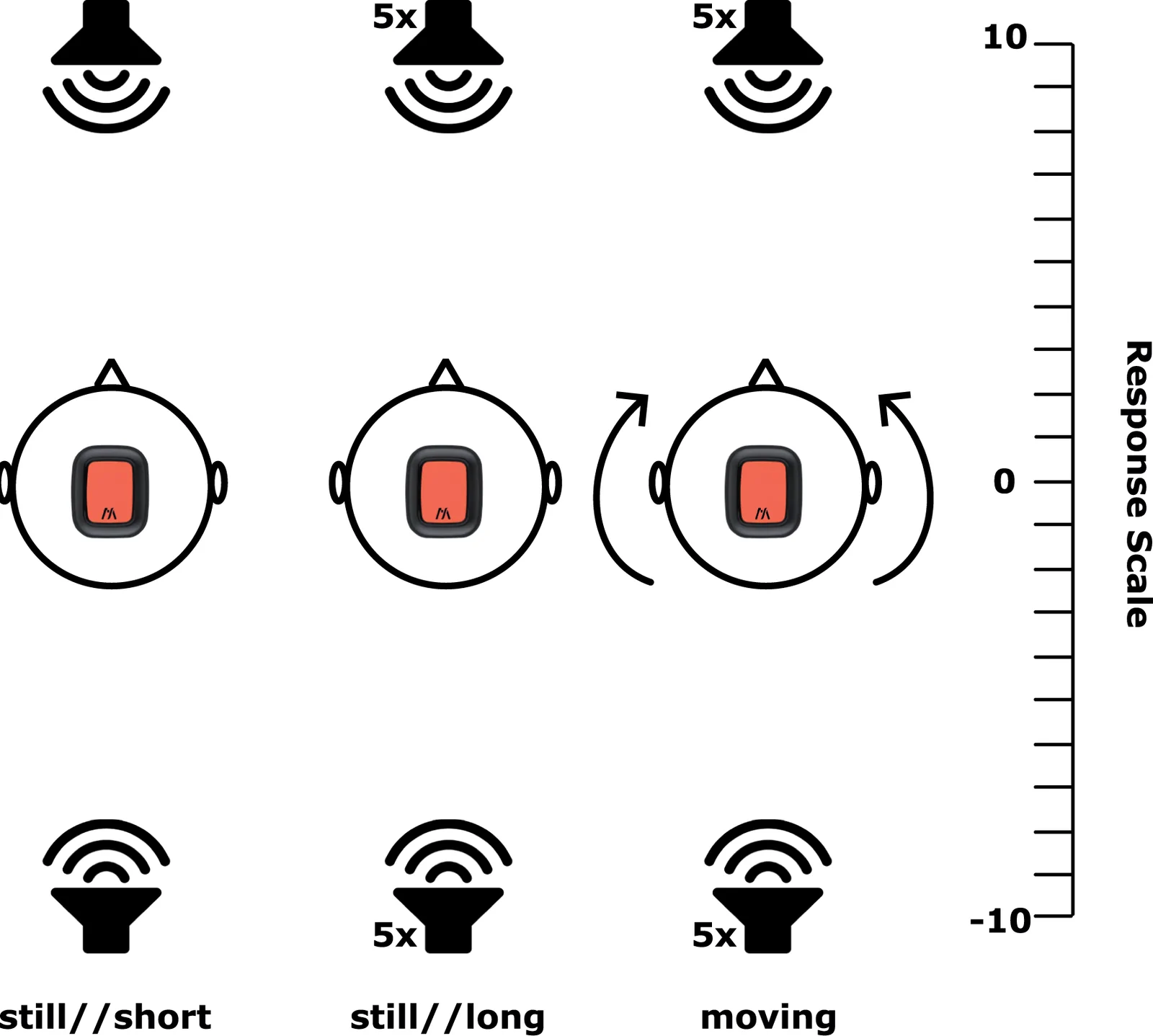
Experimental setup. Loudspeaker icons indicate stimulus presentations/repetitions. Arrows indicate the instruction for lateral head movements. On each trial, participants gave a rating in the range of ± 10, with 0 representing the center of their head

Three combinations of head movement and stimulus durations (still//short, still//long, moving//long), henceforth HM conditions, were tested under three HA conditions (unaided, BTE, ITE) for a total of nine conditions. These conditions were tested in separate blocks and the order of blocks was randomized across participants. Within a block, the two locations and three presentation levels were each tested 8 times for a total of 48 trials. In ’short’ blocks, the word was only played once. In ’long’ blocks, the word was repeated five times, with 0.5 s of silence between each repetition. While the ’short’ condition was directly adopted from Best and Roverud (2024), the ’long’ condition was included to allow ample time for head movements during stimulus presentation. For this reason, moving//short was not included as a feasible experimental condition. During the ’still’ conditions, participants were asked to keep their head and gaze fixated on a fixation cross displayed slightly above the frontal loudspeaker. No specific measures were taken to stabilize the head, but the head position was continuously tracked to confirm compliance. In the ’moving’ condition, participants were instructed to start every trial oriented towards the frontal position, then move their head laterally (left to right or right to left as if shaking the head “no”) during the five stimulus repetitions and finally return to the initial frontal position to give their response. Participants were asked to give a rating of perceptual externalization on a visual analogue scale made up of the integers from -10 to +10, with -10 representing the rear loudspeaker, 0 representing the middle of their head, and +10 representing the frontal loudspeaker. Figure 1 illustrates the experimental setup together with the response scale. To avoid confusion, the experimenter explained to participants the concept of externalization as the degree of ’out-there-ness’ as opposed to ’inside-the-head-ness’ while providing a visual representation of the scale, including loudspeakers, on paper.

### 2.4 Data Analysis

#### 2.4.1 Head Tracking

**Figure 2.**
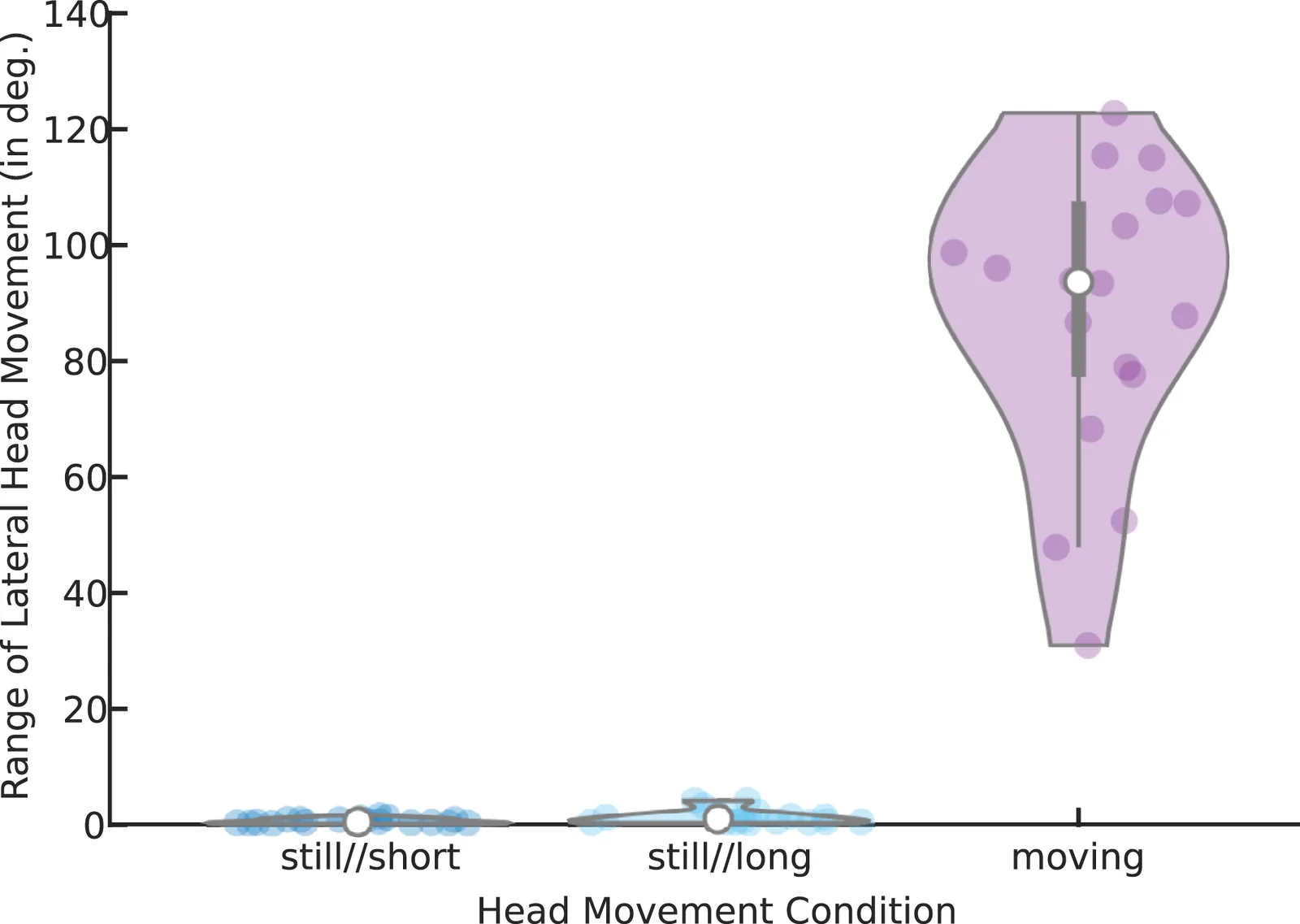
Distribution of lateral head movement range across HM conditions. Listeners (N=18) conformed well with the given instructions and made close to no lateral movements in the still conditions and larger movements in the moving condition. The median for each condition is indicated via open circles and individual subjects via closed circles. The data was averaged over all trials in all HA conditions

Head motion data was examined using MATLAB, to confirm that participants made minimal head movements during the ’still’ conditions and made reliable head movements during the ’moving’ condition. First, we manually inspected the head traces for each individual trial and removed any trials that showed evidence of technical recording issues from further analysis. This led to the exclusion of a full block of recordings (i.e. 48 trials) from each of two participants (both ITE still//long) and 14 trials from an ITE moving block from another participant. We then extracted the minimum and maximum Euler z-coordinates within any given trial to calculate the yaw rotation range. Finally, we compared these ranges across conditions using group-level summary statistics. Listeners conformed well with the given instructions (see Figure 2), showing little to no movement in the still conditions and a broader range of movement in the moving condition. On average, collapsed over HA conditions, in the still//short and still//long conditions listeners moved their head laterally 0.72° ± 0.41° and 1.49° ± 1.25°, respectively. This range of motion increased to 87.96° ± 24.94° in the moving condition.

#### 2.4.2 Behavioral Models

The behavioral data were analyzed by means of mixed-effects models. Firstly, for the externalization data, the absolute value of a listener’s scale response was the variable of interest. Secondly, for the discrimination data, the sign of each response was transformed into a binary front-back match/no-match variable of interest. Any zero response on the response scale was considered a non-matching response in terms of front-back discrimination. Using the R (Version 4.4.1) packages lme4 (Bates et al., 2015), emmeans (Lenth, 2024) and ggplot2 (Wickham, 2016), we constructed several models with different combinations and interactions of the following predictor variables: HA (unaided vs. BTE vs. ITE), HM (still//short vs. still//long vs. moving), hemifield (front vs. back) and level (50 vs. 55 vs. 60 dB SPL) and a random intercept for the participant. This was done separately for each of our two response dimensions (externalization rating and front-back discrimination).

In order to achieve normality of the residuals for the externalization models, the data were first averaged over presentation level. The winning model was a linear mixed-effects model comprised of the main effects of HA, HM and hemifield:

(1) Externalization∼HA+HM+hemifield

The winning front-back discrimination model was a generalized linear mixed-effects model, also comprised of the main effects of HA, HM and hemifield, as well as level since averaging over it was not necessary to achieve normality of residuals:

(2) Performance∼HA+HM+hemifield+level

For both models, model selection was based on the difference in Bayesian information criterion (BIC) between each considered model alternative (see Tables 2 and 3 for an overview of the evaluated models and their parameters). Significance testing was conducted using ANOVA followed by post-hoc pairwise comparisons using estimated marginal means with Tukey’s multiplicity adjustment.

#### 2.4.3 Relationship Between Externalization and Front-Back Performance

To further investigate the relationship between externalization and front-back performance, we calculated a point biserial correlation between the two measures for each individual participant across all HA conditions, but only for the head still HM conditions. Then, we tested whether the distribution of the obtained correlation coefficients was significantly different from zero using a two-tailed, one-sample t-test, implemented in MATLAB. To illustrate the relationship on a group level, we also fitted a simple linear regression model to the group-level data.

### 2.5 Relationship Between Head Movement Range and Externalization

We additionally aimed to investigate whether the actual range participants moved their heads during the moving condition had any additional effect on externalization ratings. To that end, we calculated Pearson correlation coefficients between the two measures for each individual participant and subsequently tested whether their distribution was significantly different from zero, using a two-tailed, one-sample t-test, implemented in MATLAB. On the group level, we again fitted a simple linear regression model.

## 3 Results

### 3.1 Externalization Ratings

**Figure 3.**
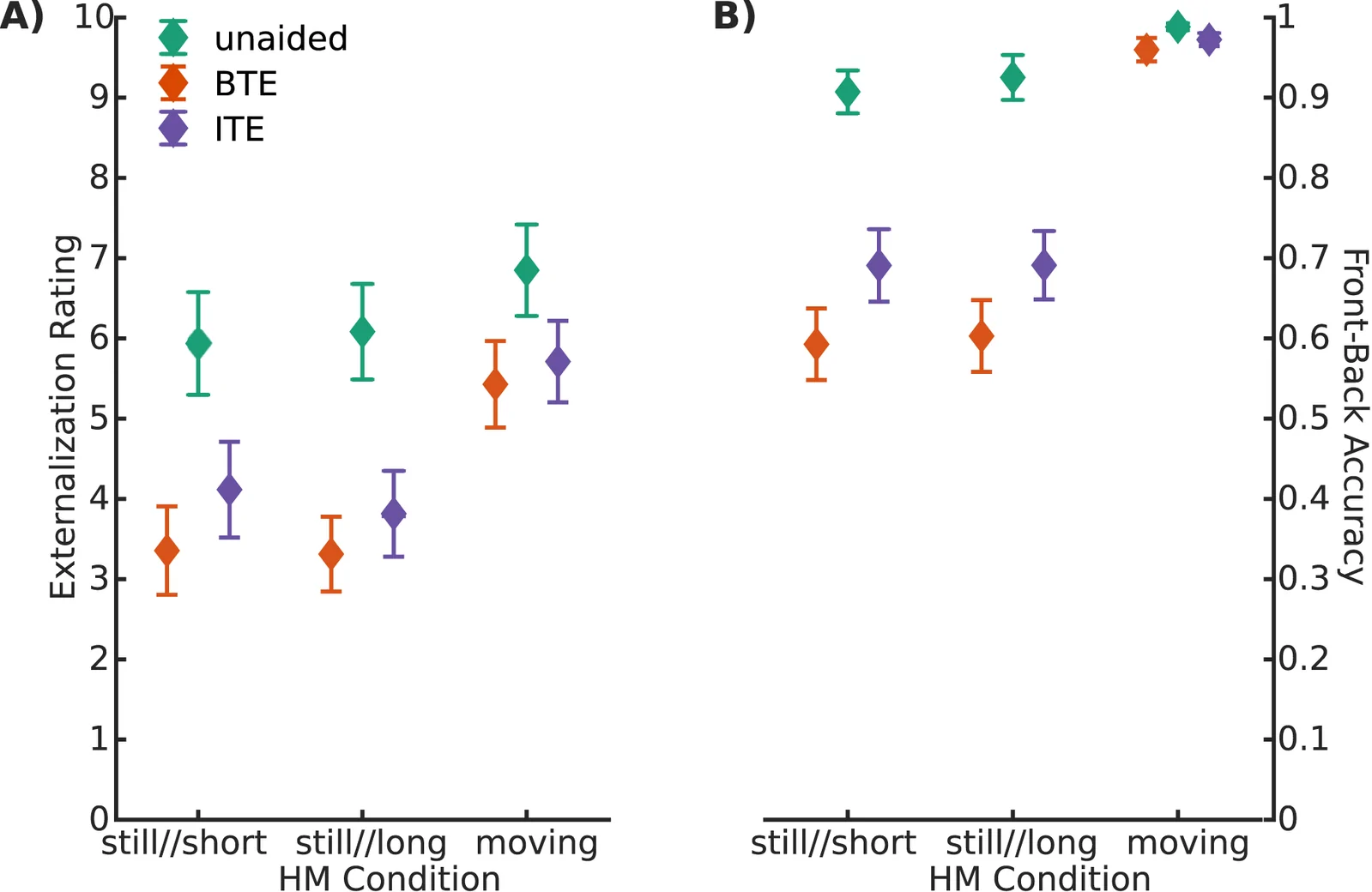
Group-Level externalization ratings and front-back discrimination Performance. (A) Listeners (N = 18) rated stimuli as most externalized in the unaided (teal) conditions and systematically as less externalized in the BTE (orange) and ITE (purple) HA conditions. There was no difference between the ’still//short’ (left column) and ’still//long’ (middle column) HM conditions, but an increase in externalization in the ’moving’ (right column) condition for all HAs conditions. (B) Listeners performed notably better without HAs than with BTE and ITE HAs in the head still conditions (left and middle column). In the ’moving’ condition, performance for all HA conditions was at, or close to, ceiling. Symbols indicate the mean and errorbars the standard error of the mean (SEM)

**Figure 4.**
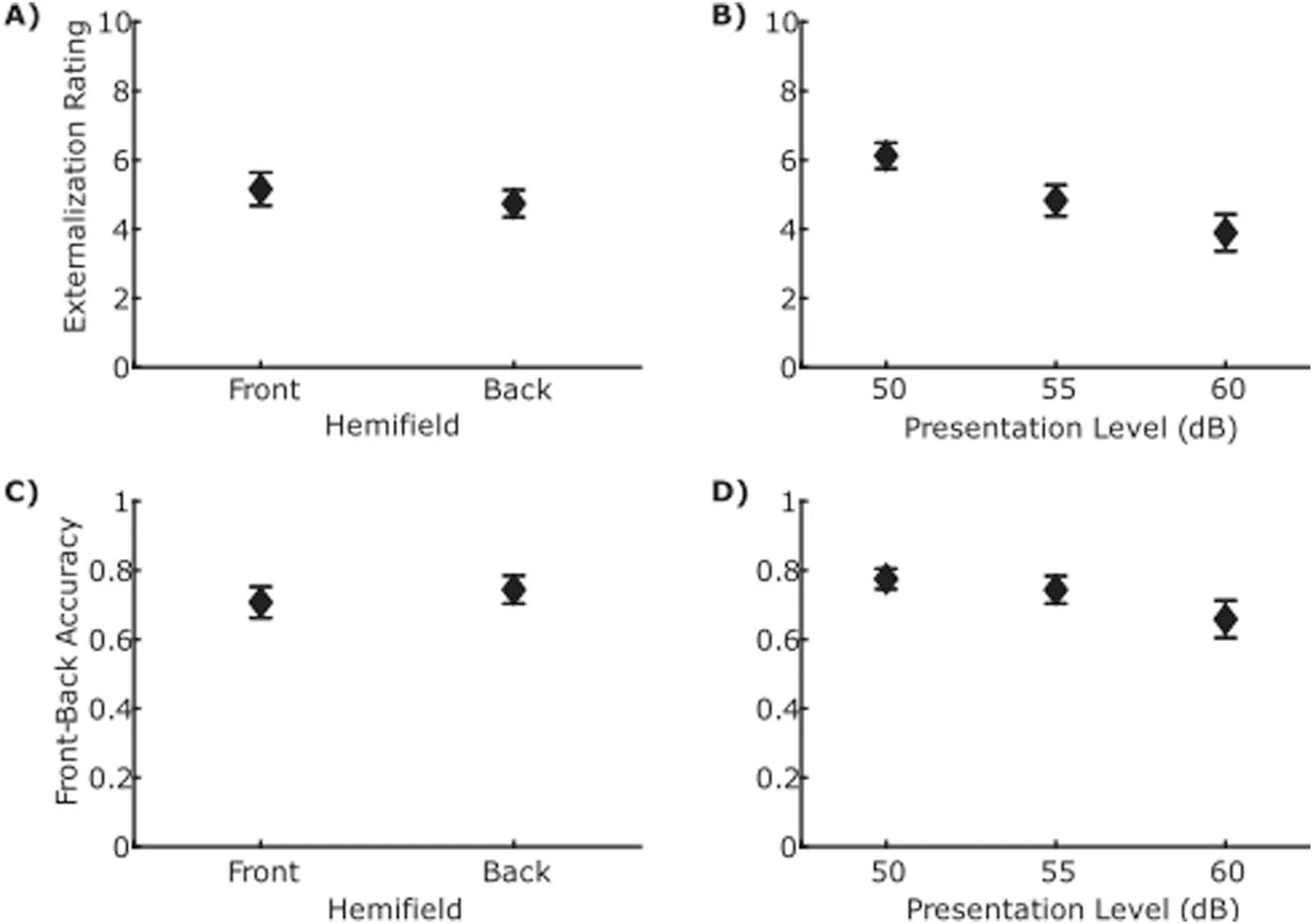
Effects of hemifield and presentation level on externalization ratings and front-back discrimination performance. (A) Listeners (N = 18) rated stimuli presented from the front as slightly more externalized than stimuli presented from the back. (B) Externalization Ratings decreased with increasing presentation level. (C) Listeners were able to discriminate better when stimuli were presented from the back. (D) Front-Back discrimination performance decreased with increasing presentation level

Figure 3A illustrates the effect of HM on externalization ratings for each HA condition. We observed the largest externalization ratings under unaided, head-moving conditions and the lowest ratings under aided, head-still conditions. The model results are shown in Table 1A, revealing significant main effects of HA, HM and hemifield. Regarding the main effect of HA, the model coefficients indicate that, in comparison to the unaided condition, both BTE and ITE conditions exhibited significantly reduced externalization ratings (unaided - BTE = 2.26, t = 10, *p* < 0.01; unaided - ITE = 1.73, t = 7.66, *p* < 0.01). There was no significant difference between BTE and ITE conditions (BTE - ITE = -0.53, t = -2.34, *p* > 0.05) but a trend towards the BTE conditions exhibiting slightly lower ratings than the ITE conditions emerged. Regarding the main effect of HM, we did not observe a significant difference between the still//short and the still//long conditions (still//short - still//long = 0.07, t = 0.29, *p* = 0.96). Between the still//short and the moving condition there was a significant difference (still//short - moving = -1.51, t = -6.65, *p* < 0.01), as was between the still long and moving condition (still//long - moving = -1.57, t = -6.94, *p* < 0.01). Regarding the main effect of hemifield, stimuli presented from the front were slightly more externalized than stimuli presented from the back (Front - Back = 0.42, t = 2.28, *p* < 0.05, see Figure 4A. The main effect of presentation level is shown in Figure 4B and a summary of post-hoc pairwise comparisons can be found in Table 4.

**Table 1.** Significance Testing for Effects of Model Predictors. (A) Results For the Externalization Model. (B) Results For the Front-Back Discrimination Model

Type II analysis of variance table					
Factor	SS	Mean sq.	df (num/den)	F value	p value
A) Externalization Ratings					
Hearing aid	303.08	151.54	2/306	55.59	< .001
Head movement	170.84	85.42	2/306	31.33	< .001
Hemifield	14.31	14.31	1/306	5.25	0.02

Factor	df	*χ*^2^ value	p value
B) Front-Back Discrimination			
Hearing aid	2	660.36	< .001
Head movement	2	778.86	< .001
Hemifield	1	20.12	< .001
Level	2	143.47	< .001

### 3.2 Front-Back Discrimination

Figure 3B illustrates the effect of HM condition on the proportion of correct front-back responses separately for each HA. The model results are shown in Table 1B, showing significant main effects of HA, HM, hemifield and level. In comparison to the unaided condition, both BTE and ITE conditions showed significantly reduced discrimination performance (unaided/BTE = 10.76, z = 25.50, *p* < 0.01; unaided/ITE = 6.34, z = 20.01, *p* < 0.01). We also observed a significant difference between the BTE and ITE conditions (BTE/ITE = 0.59, z = -7.35, *p* < 0.01). Regarding HM, the still//short condition did not differ significantly from the still//long condition (still//short/still//long = 1, z = 0.01, *p* = 1) but there was significantly better performance in the moving condition compared to both still conditions (still//short/moving = 0.07, z = -26.24, *p* < 0.01; still//long/moving = 0.07, z = -26.23, *p* < 0.01). Regarding the main effect of hemifield, participants performed slightly better when stimuli were presented from the back (Front/Back = 0.75, z = -4.49, *p* < 0.01, see Figure 4C. Regarding presentation level, the main effect is shown in Figure 4D. The model coefficients suggest significant negative linear (z = -11.48, *p* < 0.01) and quadratic (z = -2.86, *p* < 0.01) effects, indicating that performance degraded with increasing presentation level. A summary of post-hoc pairwise comparisons can be found in Table 5.

### 3.3 Relationship Between Externalization and Front-Back Performance

Qualitatively comparing the magnitude of the fixed effects between the externalization model and the front-back discrimination model (see Tables 2 and 3) reveals that most variables had a comparable effect on both response dimensions (e.g. Externalization ∼aid=BTE = -2.26; Performance ∼aid=BTE = -2.38) and that the direction was generally also the same, except for the effect of hemifield: externalization was a bit worse for stimuli presented from the back (*Externalization* ∼ *hemi* = *back* = −0.42), but discrimination performance was actually a bit better (*Performance* ∼ *hemi* = *back* = 0.29). Another notable difference is the influence of active head movements. Both models indicate positive relationships, however, the head=moving condition had a larger influence on front-back discrimination than on externalization ratings (*Performance* ∼ *head* = *movinglong* = 2.68; *Externalization* ∼ *head* = *moving* = 1.51).

**Figure 5.**
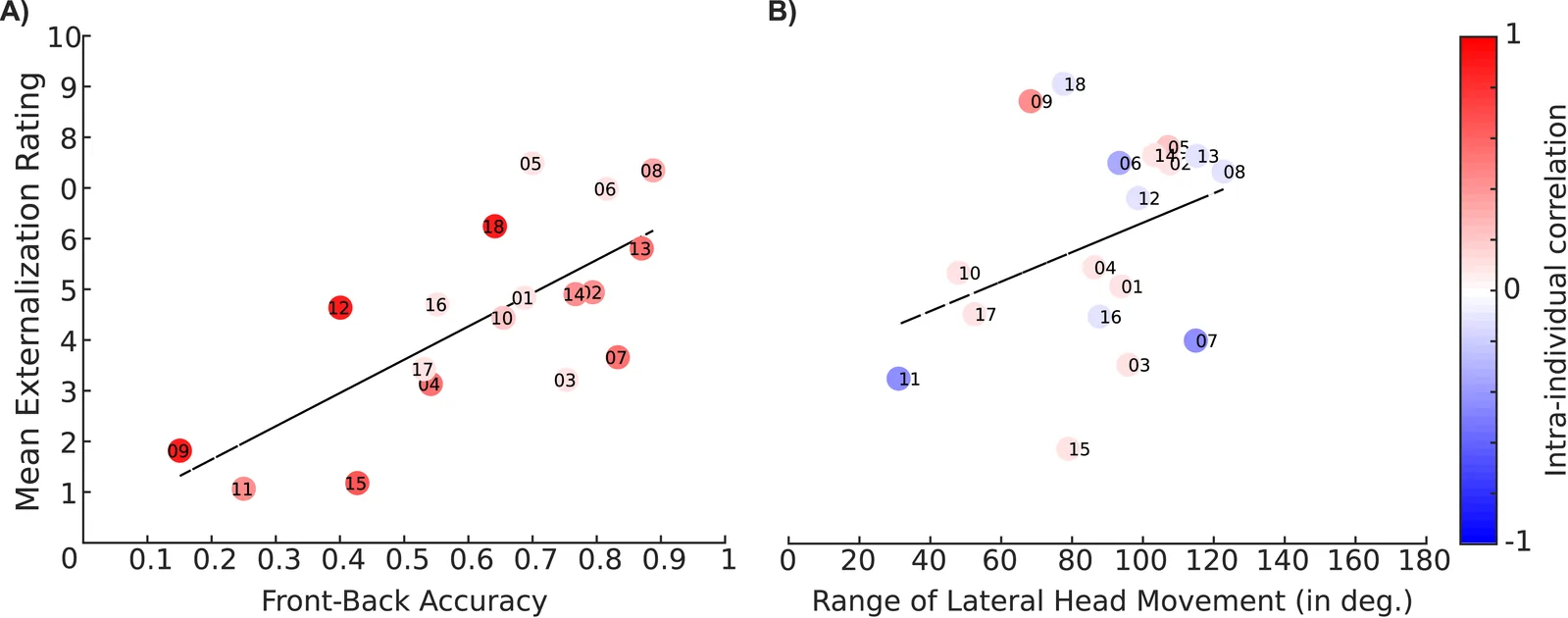
Relationship between externalization, front-back discrimination and head movement range. (A) Relationship between externalization and front-back discrimination. Each data point represents an individual participants data, averaged over the head still conditions, labeled by participant number. Red hue indicates the magnitude of a participant’s correlation between front-back performance and externalization ratings. The black line represents the fitted simple linear regression: *y* = 6.53 × *x* + 0.35. (B) Relationship between externalization and head movement range in the moving condition. Red hue indicates a positive correlation and blue hue a negative correlation. The black line represents the fitted simple linear regression: *y* = 0.03 × *x* + 3.42

Figure 5A) illustrates the relationship between externalization ratings and front-back discrimination performance both within and across participants; in the head still conditions only. A simple linear regression analysis using MATLAB’s regress function revealed that externalization ratings were positively related to front-back discrimination performance on the group level (*R*^2^ = 0.51, *F* = 16.89, *p* < 0.01). The correlation coefficients over every trial between externalization and front-back performance within participants were, on average, 0.42 (± 0.26) and their distribution was significantly different from zero (*t* = 6.77, *p* < 0.01), indicating a weak to moderate positive relationship between the two measures. Note, however, the large variance (± 0.26) and range (0.81) in correlation coefficients; an indication that there are substantial inter-individual differences. Furthermore, the correlation coefficients for two participants (01 and 06) were not significant (*p* > .05). In summary, although we do observe a significant positive correlation on the group level, no clear pattern emerges that would allow for clustering. Thus, we decided to analyze participant’s individual response strategies and patterns in more detail.

### 3.4 Inter-individual Differences

**Figure 6.**
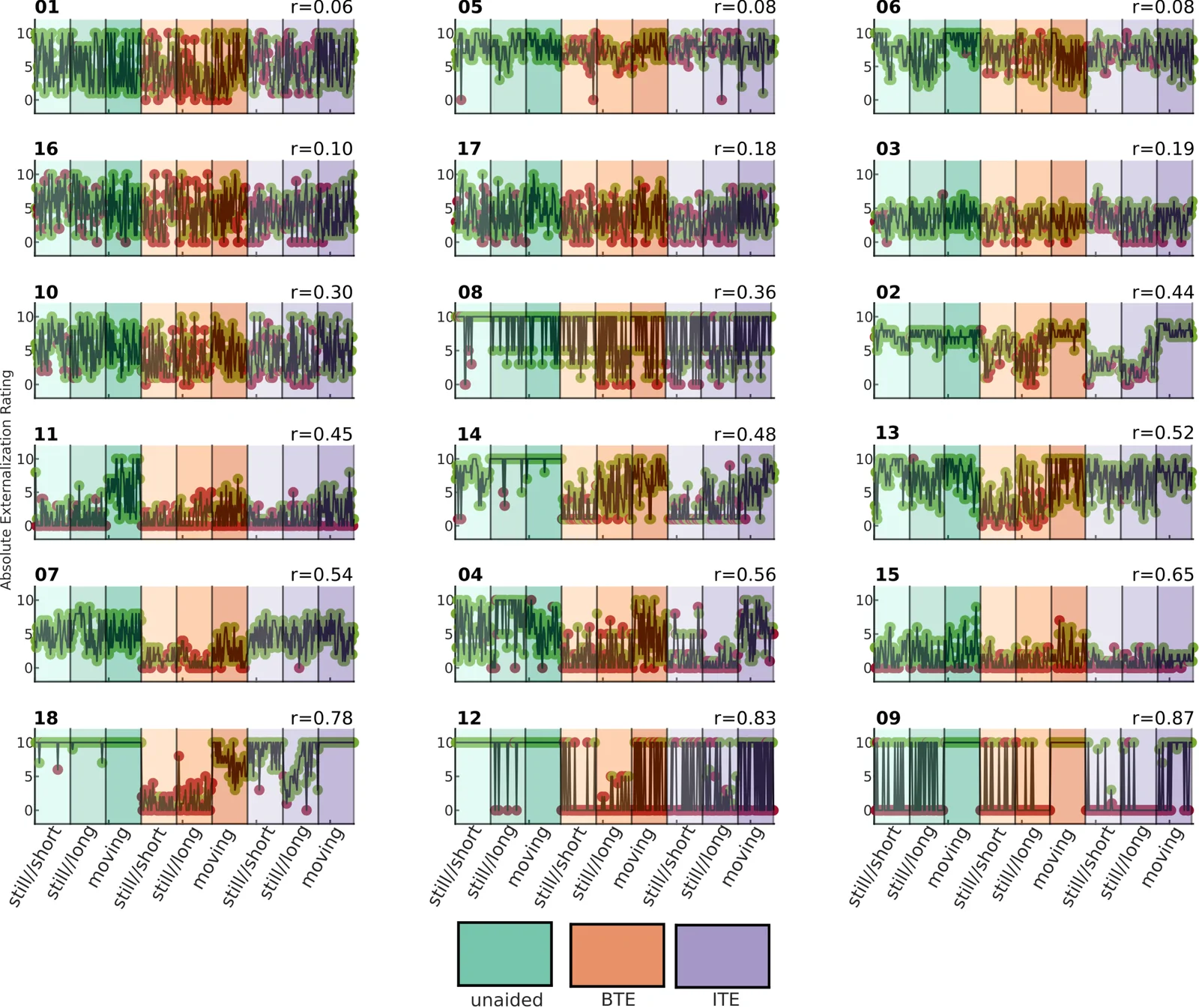
Trial-by-trial response data for all participants. The data are sorted by ascending coefficients of the correlation between externalization and front-back accuracy in the head still conditions. Each subplot represents an individual participant. Each green/red point represents an individual trial, in the order they were presented within a block. Green indicates F-B Hit and red indicates a F-B Miss. Note that a zero response is always counted as a Miss. HA conditions are represented by background color, as indicated by the color matrix legend at the bottom: teal = unaided; orange = BTE; purple = ITE. HM conditions are represented by background saturation: least saturated = still//short; semi saturated = still//long; most saturated = moving. Block order was randomized in the experiment but is here shown in the same order for each participant for visual clarity

Figure 6 shows the response patterns for all individual participants, sorted by ascending correlation coefficients. Interestingly, this sorting seems to cluster different response strategies that are discussed below using example cases.

Some participants’ externalization ratings were seemingly unaffected by front-back ambiguity and instead seemed to be driven by other factors, leading to good externalization even under conditions with strong front-back ambiguity. For instance, Participant 01 (Figure 6) was one of two participants that exhibited no significant correlation (*r* = 0.06, *p* > 0.05) between externalization ratings and front-back discrimination performance, suggesting that they may have relied on largely separate cues for both response dimensions.

As an example for moderate correlations, Participant 14 (*r* = 0.56, *p* < 0.01) exhibited clear effects of HA and HM on both externalization ratings and front-back performance. Errors cluster towards the lower half of the scale, suggesting that a breakdown in externalization was associated with front-back errors to a moderate degree.

Finally, as an example for very high correlation between externalization and front-back performance, Participant 12 (*r* = 0.83, *p* < 0.01) mostly rated externalization as a binary percept, i.e., either completely internalized (0) or perfectly externalized (±10). They made few front-back errors when the sound was successfully externalized but were mostly unable to distinguish front from back in conditions where externalization broke down, indicated by the clustering of misses towards the lower half of the scale, suggesting that they might have relied on similar cues for both judgments.

### 3.5 Relationship Between Head Movement Range and Externalization

Figure 5B) illustrates the relationship between head movement range and externalization ratings within and across participants in the head moving conditions. A simple linear regression indicates no significant relationship between the two measures on the group level (*R*^2^ = 0.12, *F* = 2.22, *p* = 0.16). The correlation coefficients within participants were, on average, 0.01 and not significantly different from zero (*t* = 0.14, *p* = 0.89). Looking at the individual correlation coefficients, however, again highlights large inter-individual differences: for some participants, externalization ratings increased with more extensive movements (e.g. 09 and 05), whereas others showed a negative correlation (e.g. 07 and 11) or barely any correlation at all (e.g. 02 and 08).

## 4 Discussion

The present study set out to investigate the potential link between front-back ambiguity and the breakdown of externalization perception in the context of HAs. We aimed to discern whether front-back ambiguity and externalization breakdown were direct consequences of one another, wholly independent, or partially related. We found evidence supporting front-back ambiguity as a potential contributing factor to the breakdown of externalization that can occur when listening through HAs. At the group level, resolving front-back ambiguity with head movements substantially improved externalization but did not fully restore the percept to unaided levels, suggesting that front-back ambiguity contributes to breakdowns of externalization but is not the only cause. Given that the direction of causality is not known, another way of interpreting our findings is that improvements in externalization through head movements can fully resolve front-back ambiguity. Alternatively, it may be that head movement provides cues (e.g., dynamic binaural cues) that independently improve externalization and resolve front-back ambiguity. At the individual level, we observed substantial inter-individual differences regarding the relationship between front-back ambiguity and perceptual externalization, suggesting highly individual perceptual decision making. In the following, we first discuss the effects of HAs on externalization and front-back discrimination, then reflect on the additional influence of head movements, and finally discuss reasons and limitations for the relationship between the two perceptual outcomes at the group and individual levels.

### 4.1 Hearing Aids Disrupt Externalization and Front-Back Discrimination

Since our experimental design closely followed Best and Roverud (2024) and replicated the NH ”unaided”, ”BTE closed” and ”ITE closed” conditions in their front-back setup (here: ”Unaided still//short”, ”BTE still//short” and ”ITE still//short”), a direct comparison is possible. Regarding externalization ratings, we observed average ratings of around 6 for unaided and of around 3.5 for BTE, consistent with Best and Roverud (2024). Ratings for the ITE conditions in the present study were a bit lower (around 5 in Best and Roverud (2024) and around 4 here), likely explaining the absence of a significant difference between the BTE and ITE conditions, although the overall pattern of the data is similar (i.e. slightly lower ratings for the BTE, compared to the ITE condition). In terms of front-back discrimination, both studies show similar patterns. We were able to replicate close-to-ceiling unaided performance and significantly worse performance in both HA conditions. Furthermore, we also found a significant difference between BTE and ITE conditions, although performance was slightly poorer in the current study, especially for the ITE condition, resulting in a less pronounced difference. In summary, our data support the main conclusions drawn by Best and Roverud (2024): HAs disrupted perceptual externalization and front-back discrimination, and this effect was more pronounced for BTE HAs compared to ITE HAs.

Also in line with Best and Roverud (2024), in the unaided condition externalization ratings were quite restricted, i.e. unaided stimuli were not rated as perfectly externalized. This was the case even when head movement was introduced, which is surprising as incidental translational lateral movements in the moving condition may have provided additional motion parallax cues to distance (Genzel et al., 2018). There are several possible reasons for the restricted externalization ratings that we routinely observe under “ideal” conditions. For instance, the lack of realistic reverberation in the experimental booth. Sounds exhibiting realistic reverberation have been shown to more likely be externalized compared to anechoic sounds (Best et al., 2020). In the current study the experimental booth provided little reverberation and may thus not have been representative of the real-world environments making up the usual listening experience of our participants. Introducing more realistic reverberation could lead to a significant improvement in externalization perception overall and perhaps a more robust percept that is less sensitive to the subtle distortions introduced by HAs. Indeed, individualized spectral-shape cues have been shown to be less important for externalization perception in reverberant environments than in anechoic environments (Leclère et al., 2019; Li et al., 2020). Furthermore, providing compelling visual information that is co-ordinated with the acoustic information may also increase realism and in turn reduce a listener’s reliance on precise acoustical details. Calcagno et al. (2012) argue, for instance, that auditory distance perception is generally affected by visual information. Externalization ratings have been shown to increase when congruent visual room information, as opposed to no visual information, is provided to listeners (Udesen et al., 2015; Werner et al., 2016). Similarly, Gil-Carvajal et al. (2016) reported the highest degree of externalization under conditions where the audio-visual information about the recording and playback environment was congruent. Considering that the stimuli used in the current experiment were not recorded in the same environment that the experiment took place in, this could also have contributed to unaided ratings being rather low to begin with. However, recording and playback rooms were rather similar, so there likely wasn’t a huge mismatch between the two. Other visual cues, such as lip movements and facial expressions, likely also contribute to externalization perception, but the literature on this is sparse. Alternatively, participants might have perceived the sounds as fully externalized but did simply not make use of the full rating scale. As evident from Figure 6, many participants seemed hesitant to rate sounds as fully externalized or internalized, perhaps in anticipation of a subsequent sound that might be perceived as even more ’out-there’ or ’inside-the-head’.

The human auditory system is also highly adaptive and it has been shown that participants can, after an extensive training period, calibrate to distorted spectral cues (presumably by updating their established templates) for spatial hearing (Mendonça, 2014). However, Mendonça et al. (2013) noted that externalization improved at a slower rate than directional localization, suggesting distinct learning processes. Adaptation effects introduced by prolonged and persistent HA use could thus lead to template updates and reduce the externalization breakdown compared to what we observed in our “acute” measurements. Some evidence against this idea comes from Best and Roverud (2024), who found that experienced HA users exhibited reduced externalization (relative to unaided listening) even with their own devices.

### 4.2 Head Movements Only Partially Restore Externalization

Our experiment was motivated by the idea that head movements, which introduce dynamic interaural cues that provide unambiguous information about whether a sound is coming from the front or the back, may reduce the weight that is placed on spectral-shape cues. A similar reduction of spectral-cue weighting for externalization perception has previously been demonstrated under the presence of reverberation cues (Li et al., 2020). Our results partly support this idea: externalization ratings with HAs were significantly improved when listeners moved their heads during stimulus presentation. However, the fact that ratings did not recover to unaided levels suggest that some weight was still placed on spectral cues and that the dynamic cues conveyed by HAs were not as reliable, or that there was another contributing factor breaking down externalization, such as the unnatural gain introduced by occluded ear canals (Best & Roverud, 2024). Past work has argued that non-linear HA processing can distort dynamic binaural cues and thus impair self-motion processing (Brimijoin & Akeroyd, 2016). Even though the present study used a linear gain across all frequencies, some binaural artifacts, such as differences in occlusion between the left and right ear canal, could have lead to similar effects.

### 4.3 Inter-individual Differences

Finally, we observed a significant positive correlation between externalization and front-back performance at the group level for the head still conditions. However, this relationship was not present in all participants when examined individually, as illustrated in Figure 5. While past work has similarly highlighted inter-individual differences both in front-back discrimination (Wenzel et al., 1993; Wightman & Kistler, 1989; Zhang & Hartmann, 2010) and in externalization/distance perception (Baumgartner et al., 2017; Catic et al., 2015; Hartmann & Wittenberg, 1996), the present study suggests that these individual differences do not have a common basis across the two dimensions.

It should be noted again that, for the present analysis, any zero externalization response was considered a non-matching front-back discrimination response. This likely inflated the correlation coefficients for participants who exhibited a more binary response pattern and, thus, strengthened the clustering of response strategies based on correlation coefficients. An approach comparing (i.e. correlating) the coefficients of the separately fitted models instead of the actual data points would have allowed for a more detailed analysis of how individuals may have weighted different factors. This was not possible, as we only had model coefficients on the group level.

Nonetheless, our results suggest highly individualized response strategies, which we speculate could have arisen due to a number of factors. For example, physiological factors such as individual ear morphology likely contributed to the amount of occlusion participants experienced in the HA conditions (Cubick et al., 2018), leading to differences in terms of cue availability. Participants with larger ear canals may have experienced less occlusion, which in turn could have improved externalization perception. As Best and Roverud (2024) noted, conditions with lower amounts of occlusion (i.e. open HA domes) also exhibited higher externalization ratings. The specific fit may have also affected the effective gain, resulting in variations in the received spectrum and level. Alternatively, more cognitive factors such as individual weighting of visual and auditory cues could have also contributed to distinct response patterns. Some participants’ responses were likely more driven by factors mostly unaffected by HA and HM, such as presentation level and hemifield. In addition, participants who almost exclusively gave binary externalization responses may have been heavily biased by their prior knowledge about the actual loudspeaker positions, whereas others were not.

### 4.4 Limitations and Outlook

Our data is limited to NH listeners, making it difficult to generalize our results to HI listeners whose auditory system might interact differently with disruptions introduced by HAs. Furthermore, the scale used in the present study implicitly confounds externalization with distance perception. Recent work has argued that, even though these two aspects of spatial hearing are correlated, they are likely distinct aspects of spatial hearing because they have been shown to be affected differently by a number of auditory cues such as reverberation, ITDs and ILDs, and sound type (Lavandier et al., 2024). Thus, effects we report could also be attributed to distance perception rather than externalization. Some authors also argue that externalization perception is a binary decision, i.e. a sound is either externalized or not, whereas others instead argue that it constitutes more of a continuous percept (see Best et al. (2020)). Interestingly, the data presented in the current study reflects this ongoing discussion. It seems that for some participants, externalization truly was a binary percept, whereas others clearly experienced a continuum of percepts.

Informal reports from our participants revelead that the externalization percept could even change during the stimulus presentation, presumably reflecting the dynamic acoustics of speech. Thus, a more interactive measure, i.e. a representation where changes in externalization can be tracked/indicated in real-time, would be useful for a more nuanced understanding of externalization perception. In general, alternative response methods may also circumvent possible biases in the integer-based subjective rating scale and more accurately capture each participant’s perceptual experience. Similarly, future work should address whether the introduction of additional congruent and incongruent, audio-visual cues alters the effects we report here.

## Supplemental Material

Supplemental Material - The Effect of Head Movements on Front-Back Discrimination and Sound Externalization With Hearing Aids Supplemental Material for The Effect of Head Movements on Front-Back Discrimination and Sound Externalization With Hearing Aids by Tobias Greif, Virginia Best, Robert Baumgartner in Trends in Hearing

## Data Availability

The data that support the findings of this study are available from the following repository: DOI: 10.17605/OSF.IO/SFA2J.
