# Supplementary material for: The Effect of Head Movements on Front-Back Discrimination and Sound Externalization With Hearing Aids
Source: Trends Hear. 2026 Aug 27;30:23312165261480035. doi: 10.1177/23312165261480035 (PMC13525295; doi:10.1177/23312165261480035)
Supplement: Supplementary file 1 [file sj-pdf-1-tia-10.1177_23312165261480035.pdf]

Appendix

**Models**	**dBIC**
**1.** $Ext \sim (aid + head + hemifield)\|sbj$	0
**2.** $Ext \sim (aid * head + hemifield)\|sbj$	14.97
**3.** $Ext \sim (aid * head * hemifield)\|sbj$	54.9
**4.** $Ext \sim (aid)\|sbj$	n.a.
**5.** $Ext \sim (head)\|sbj$	n.a.
**6.** $Ext \sim 1\|sbj$	n.a.

**Fixed effects coefficients (model 1)**	**Est**	**SE**	**t**	**Pr**$(>\|t\|)$	
(Intercept) reference aid=unaided, head=still//short, hemifield=front	6.01	0.46	12.94	< 0.01	***
aid=BTE	-2.26	0.22	-10.08	< 0.01	***
aid=ITE	-1.73	0.22	-7.72	< 0.01	***
head=still//long	-0.07	0.22	-0.29	0.77	
head=moving	1.51	0.22	6.70	< 0.01	***
hemifield=back	-0.42	0.18	-2.29	0.02	*

**Predictions (model 1) for Ext**	**Head:**	**still//short**	**still//long**	**moving**
aid=unaided, hemifield=front		**6.01**	**5.95**	**7.52**
aid=unaided, hemifield=back		**5.59**	**5.53**	**7.10**
aid=BTE, hemifield=front		**3.75**	**3.68**	**5.25**
aid=BTE, hemifield=back		**3.33**	**3.26**	**4.83**
aid=ITE, hemifield=front		**4.28**	**4.21**	**5.78**
aid=ITE, hemifield=back		**3.86**	**3.79**	**5.36**

Table 2. Externalization model summary. Several models are estimated and compared relative to the winning model #1 in terms of BIC difference. Smaller BIC values indicate better model fit. For the selected model #1, fixed effects are reported with model estimates (Est), standard errors (SE), t-values, corresponding p-values and model predictions. Fixed effects are relative to the respective reference levels (unaided for aid, still//short for head). Ext = absolute externalization rating (0-10); aid = HA condition (unaided, BTE, ITE); head = HM condition (still//short, still//long, moving); hemifield = loudspeaker position (Front, Back); sbj = participant ID (1-18).

Models	dBIC
**1.** $Performance \sim (aid + head + hemifield + level)\|sbj$	0
**2.** $Performance \sim (aid * head + hemifield + level)\|sbj$	31.39
**3.** $Performance \sim (head)\|sbj$	958.67
**4.** $Performance \sim (aid)\|sbj$	1264.68
**5.** $Performance \sim (level)\|sbj$	1870.38
**6.** $Performance \sim (hemifield)\|sbj$	n.a.
**7.** $Performance \sim (aid * head * hemifield + level)\|sbj$	n.a.

Fixed effects coefficients (model 1)	Est	SE	z	Pr($>\|z\|$)	
(Intercept) reference aid=unaided, head=still//short, hemifield=front	2.02	0.29	6.97	< 0.01	***
aid=BTE	-2.38	0.09	-25.5	< 0.01	***
aid=ITE	-1.85	0.09	-20.01	< 0.01	***
head=still long	-0.01	0.07	-0.01	0.99	
head=moving long	2.68	0.10	26.24	< 0.01	***
hemifield=back	0.29	0.06	4.49	< 0.01	***
level=linear	-0.64	0.06	-11.48	< 0.01	***
level=quadratic	-0.16	0.06	-2.86	< 0.01	**

Predictions (model 1) for performance	Head:	still//short	still//long	moving
aid=unaided, hemifield=front		**0.88**	**0.88**	**0.99**
aid=unaided, hemifield=back		**0.90**	**0.90**	**0.99**
aid=BTE, hemifield=front		**0.41**	**0.41**	**0.91**
aid=BTE, hemifield=back		**0.48**	**0.48**	**0.93**
aid=ITE, hemifield=front		**0.54**	**0.54**	**0.95**
aid=ITE, hemifield=back		**0.61**	**0.61**	**0.96**

Table 3. Front-back discrimination model summary. Several models are estimated and compared relative to the winning model #1 in terms of BIC difference. Smaller BIC values indicate better model fit. For the selected model #1, fixed effects are reported with model estimates (Est), standard errors (SE), z-ratios, corresponding p-values and model predictions. Fixed effects are relative to the respective reference levels (unaided for aid, still//short for head). Performance = match (0, 1); aid = HA type (unaided, BTE, ITE); head = HM condition (still//short, still//long, moving); hemifield = loudspeaker position (Front, Back); sbj = participant ID (1-18).

Externalization Model: Pairwise Comparisons

A) Pairwise Comparisons for HM

contrast	estimate	SE	df	t ratio	p value
still//short - still//long	0.07	0.23	311	0.29	0.96
still//short - moving	-1.51	0.23	311	-6.65	$< .001$
still//long - moving	-1.57	0.23	311	-6.94	$< .001$

B) Pairwise Comparisons for HA

contrast	estimate	SE	df	t ratio	p value
unaided - BTE	2.26	0.23	311	10	$< .001$
unaided - ITE	1.73	0.23	311	7.66	$< .001$
BTE - ITE	-0.53	0.23	311	-2.34	> 0.05

C) Pairwise Comparisons for hemifield

contrast	estimate	SE	df	t ratio	p value
Front - Back	0.42	0.19	311	2.28	$< .005$

Table 4. Post-hoc pairwise comparisons for externalization. P values were adjusted using Tukey's method for comparing a family of three estimates.

Front-Back Discrimination Model: Pairwise Comparisons

A) Pairwise Comparisons for HM

contrast	odds ratio	SE	df	z ratio	p value
still//short / still//long	1.0	0.07	Inf	0.001	1
still//short / moving	0.07	0.02	Inf	-26.24	$< .001$
still//long / moving	0.07	0.02	Inf	-26.24	$< .001$

B) Pairwise Comparisons for HA

contrast	odds ratio	SE	df	z ratio	p value
unaided / BTE	10.76	1.00	Inf	25.50	$< .001$
unaided / ITE	6.34	0.58	Inf	20.01	$< .001$
BTE / ITE	0.59	0.04	Inf	-7.35	$< .001$

C) Pairwise Comparisons for hemifield

contrast	odds ratio	SE	df	z ratio	p value
Front / Back	0.75	0.05	Inf	-4.49	$< .001$

D) Pairwise Comparisons for level

contrast	odds ratio	SE	df	z ratio	p value
50dB / 55dB	1.30	0.11	Inf	3.21	$< .001$
50dB / 60dB	2.49	0.20	Inf	11.48	$< .001$
55dB / 60dB	1.92	0.15	Inf	8.41	$< .001$

Table 5. Post-hoc pairwise comparisons for front-back discrimination. Tests were performed on the log offs ratio scale and p values were adjusted using Tukey's method for comparing a family of three estimates.
